# An Isolated Splenic Hydatid Cyst

**DOI:** 10.7759/cureus.36571

**Published:** 2023-03-23

**Authors:** Hovra Zahoor, Esra Sari, Jay Patel, Nilmarie Guzman

**Affiliations:** 1 Internal Medicine, HCA Florida Orange Park Hospital, Orange Park, USA

**Keywords:** partial splenectomy, hydatid serology, hydatid, cyst hydatid, splenic cyst, echinococcosis granulosus

## Abstract

An isolated hydatid cyst of the spleen is a rare presentation of echinococcal diseases, especially in non-endemic areas where it may end up with unnecessary work-up and misdiagnosis. Here, we present the case of a 28-year-old female presenting with generalized abdominal pain, constipation, and early satiety who had a delayed diagnosis of isolated splenic hydatid cyst which was partially treated with albendazole, eventually requiring splenectomy.

## Introduction

Echinococcal disease is caused by infection with the metacestode stage of the tapeworm *Echinococcus*, which belongs to the family Taeniidae. Cystic echinococcosis (also known as hydatid cyst) is caused by *E. granulosus*, one of the most common species of *Echinococcus *[1]. Although it presents as an endemic transmission in some areas of South America, Asia, and Africa, it is rare in European countries and the United States. The disease is most commonly localized in the liver and lungs, although other organs including the kidney, bones, and brain can be affected due to systemic dissemination. This slowly progressing disease can follow an asymptomatic period even for years, and with enlargement in cyst size, patients may present with various vague symptoms (e.g., abdominal pain, generalized weakness, weight loss) depending on the affected organ system. Certain complications including cyst rupture and infection can precipitate symptoms. Other sites such as the heart, spleen, pancreas, and muscles are very rarely affected. An isolated hydatid cyst in otherwise uncommonly affected organs without evidence of systemic dissemination is rare. Here, we present the case of a 28-year-old Caucasian female with no past medical history who presented with generalized abdominal pain and constipation. She was found to have an isolated cyst in the spleen which was identified as a hydatid cyst. Our case highlights the challenges encountered with the rare presentations of hydatid cysts such as an isolated hydatid cyst in the spleen and navigating through the diagnostic steps to help establish a diagnosis for proper and safe management.

## Case presentation

A 28-year-old Caucasian female with no significant past medical history presented to our facility with a two-week history of constipation, generalized abdominal discomfort, and early satiety. She reported backpacking for seven months (almost one year before the presentation) and had been to Europe, the Balkans, and Southeast Asia. She stated that she was staying at hostels and couch surfing frequently where she had animal contact including dogs and cats. She also reported eating street food frequently. She was seen by a provider one month prior with similar symptoms and was treated with empiric antibiotics for possible pyelonephritis. She reported that her symptoms failed to improve and had since progressively worsened which prompted her to present to our institution. Upon presentation to our institution, the physical examination was unremarkable except for tachycardia.

**Figure 1:**
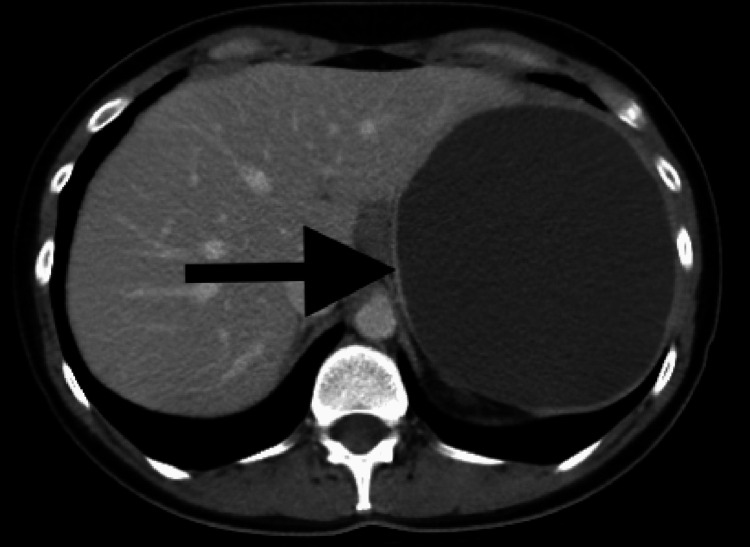
Computerized tomography (CT) of the abdomen/pelvis with intravenous contrast: a large cyst seen in the spleen measuring 11.9 × 10.6 cm in the axial dimension. Mass effect on the stomach can be seen with the partial displacement of the pancreas and kidney.

On workup, complete blood count, comprehensive metabolic panel, and liver function tests were unremarkable. She underwent computerized tomography (CT) of the abdomen/pelvis with intravenous (IV) contrast, which showed a large cyst measuring 11.9 × 10.6 cm within the spleen pushing on the stomach (Figure 1).

**Figure 2:**
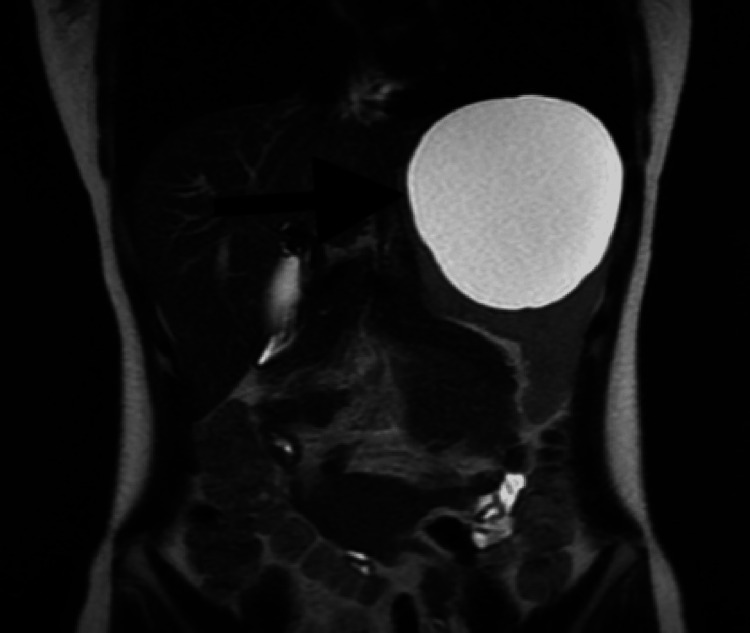
Magnetic resonance imaging of the abdomen/pelvis with and without contrast: a large splenic cyst can be seen within the left upper quadrant exerting a mass effect on the stomach.

The cyst had a benign appearance with no thickening, septa, or complex features. Subsequent CT of the chest and magnetic resonance imaging (MRI) of the brain and abdomen/pelvis with and without contrast were negative for any other cystic lesions including any liver lesions (Figure 2).

**Figure 3:**
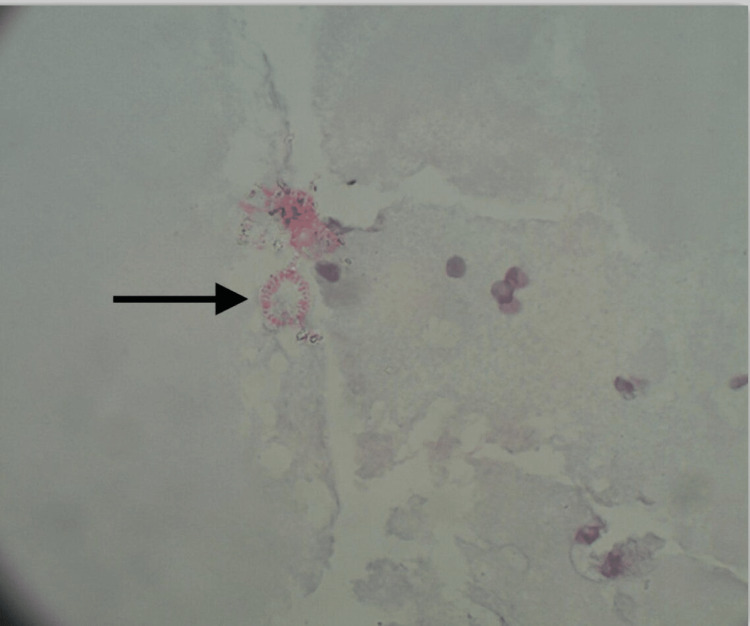
Hematoxylin and eosin stain, 100× magnification of specimen (obtained from puncture-aspiration-injection-reaspiration) revealing disintegrated round structures with occasional granules and a rarely preserved ring structure with hooklets suspicious of Echinococcus.

Despite the lack of characteristic clinical and imaging findings, we held a high index of suspicion for *Echinococcus *given the patient’s travel history and the risk of anaphylaxis if any open surgical procedure was performed in the event of a hydatid cyst. After consultation with gastroenterology and infectious disease, we sent for echinococcal serologies and initiated the patient on albendazole 400 mg twice daily. While pending echinococcal serologies, the puncture-aspiration-injection-reaspiration (PAIR) procedure was done by interventional radiology (IR) with 500 cc of serosanguinous fluid aspirated from the splenic cyst. The pathology of the aspirate revealed scattered calcareous bodies, round structures with occasional granules, and a ring structure with hooklets highly suspicious of *Echinococcus *(Figure 3).

Interventional radiology attempted a sclerosis procedure with intralesional hypertonic saline injection but the procedure was unsuccessful due to failure to aspirate distal fluid which was attributed to the chronic nature of the cyst. Echinococcal serologies remained negative during this time. Interval CT of the abdomen/pelvis with IV contrast was done which revealed worsening collection in the spleen appearing more homogenous and slightly higher in density in comparison to the prior examination. At this point, the decision was made to pursue splenectomy given treatment failure with albendazole and minimally invasive drainage attempts. Albendazole was continued during this time and the patient was transferred to another facility for an urgent splenectomy. The patient eventually underwent a splenectomy.

## Discussion

Hydatid disease can affect any organ. The most common site of disease is the liver, followed by the lungs, kidneys, bones, and brain. Other sites such as the heart, spleen, pancreas, and muscles are very rarely affected [2]. According to a systematic review, splenic echinococcal involvement develops in 1% to 3.3% of the patients [1]. Other studies report this percentage to be between 2% and 5% [3,4]. However, most reported cases had concomitant hepatic hydatidosis, showing systematic dissemination. According to a study from China, only 0.7% of 3,003 patients with cystic echinococcus had an isolated splenic involvement [5]. It is to be noted that China is one of the endemic regions for echinococcal infection. Despite an increase in its occurrence, echinococcosis remains a very rare disease in the continental United States.

The symptomatology of hydatid cysts varies given the slow progression of the disease, especially if a cyst remains uninfected/unruptured. Due to the slowly progressive nature of cyst growth, patients may remain asymptomatic for many years. Patients may present with non-specific symptoms such as abdominal pain, generalized weakness, or weight loss. Depending on the organ involvement, symptoms such as cough, nausea and vomiting, bloody stools, and chest pain may be added to the symptoms. If a cyst becomes complicated (infected or ruptured), the symptoms (depending on the organ involvement, e.g., abdominal pain, fever/chills) would be expected to be more severe. Moreover, the rupture of a cyst can lead to an allergic reaction/anaphylaxis and even death. Not infrequently, the diagnosis is incidentally established when a patient is evaluated for other diseases [6]. Our patient presented with non-specific symptoms. Her symptomatology included constipation, generalized abdominal pain, and early satiety probably related to the presence of a large abdominal mass but had been asymptomatic until a month before.

Confirmation of the diagnosis depends on imaging modalities, mostly abdominal ultrasonography and CT scan, serologies, and pathological evaluation. Calcification of the cyst wall and the presence of daughter cysts, cystic membranes, septa, or hydatid sand are imaging findings consistent with splenic hydatid disease. In most cases, serologic tests detecting anti-echinococcus antibodies and imaging characteristics in combination indicate the correct diagnosis [7,8]. Despite all the above investigations, diagnosis is always challenging. In another study, it was described that a detailed personal history and the presence of calcification, daughter cysts, or concomitant cystic lesions in the liver and other organs are helpful for the diagnosis of splenic hydatidosis [9]. Our case represents a unique diagnostic scenario. The lack of classic clinical presentation for hydatid disease and concomitant cystic lesions in other organs along with negative serologies made the diagnosis challenging in our case. There is a lack of data to deal with such diagnostic challenges. In our case, we held a high index of suspicion and used our clinical judgment to guide us through the dilemma. The diagnosis was established after the patient underwent the PAIR procedure which involves aspiration of the cyst contents via a special cannula, followed by the injection of a scolicidal agent for at least 15 minutes, and then re-aspiration of the cystic contents. The patient underwent the PAIR procedure, and the pathology of the aspirate revealed cystic structures suspicious of *Echinococcus*.

Treatment is mainly surgical and options depend on the individual patient and surgeon’s expertise. Total splenectomy, partial splenectomy, cyst enucleation, and unroofing with omentoplasty have all been reported [10]. Percutaneous drainage of the splenic hydatid cyst with injection and consecutive reaspiration of a scolicidal agent (PAIR technique) has been proposed as an alternative, non-surgical therapy for patients at high anesthetic risk or who do not agree to surgery [11]. Given the diagnostic challenge in our case, we did not opt for splenectomy as the first line for management. We proceeded with PAIR which contributed to establishing the diagnosis. Eventually, a decision for splenectomy was made.

## Conclusions

Limited data are available on epidemiology, clinical presentation, and establishing a diagnosis for an isolated splenic echinococcal cyst. Our case represents the importance of a high index of suspicion and clinical judgment in challenging diagnostic scenarios as recognition is critical given the risk of an anaphylactic reaction, potential death in the event of misdiagnosis, and open surgical or other unwarranted procedures. It also highlights the need for more research toward identifying the best diagnostic modalities in patients with isolated splenic echinococcal cysts.
